# Crystal structures of 2,2′-bipyridin-1-ium 1,1,3,3-tetracyano-2-ethoxyprop-2-en-1-ide and bis(2,2′-bipyridin-1-ium) 1,1,3,3-tetracyano-2-(dicyanomethylene)propane-1,3-diide

**DOI:** 10.1107/S2056989015007306

**Published:** 2015-04-18

**Authors:** Zouaoui Setifi, Arto Valkonen, Manuel A. Fernandes, Sami Nummelin, Habib Boughzala, Fatima Setifi, Christopher Glidewell

**Affiliations:** a Laboratoire de Chimie, Ingénierie Moléculaire et Nanostructures (LCIMN), Université Ferhat Abbas Sétif 1, Sétif 19000, Algeria; b Unité de Recherche de Chimie de l’Environnement et, Moléculaire Structurale (CHEMS), Université Constantine 1, Constantine 25000, Algeria; c Department of Chemistry, University of Jyväskylä, PO Box 35, FI-40014 Jyväskylä, Finland; d School of Chemistry, University of the Witwatersrand, PO Wits, 2050 Johannesburg, South Africa; e Molecular Materials, Department of Applied Physics, School of Science, Aalto University, PO Box 15100, FI-00076 Aalto, Finland; f Laboratoire de Matériaux et Cristallochimie, Faculté des Sciences de Tunis, Université de Tunis El Manar, 2092 Manar II Tunis, Tunisia; g School of Chemistry, University of St Andrews, Fife KY16 9ST, Scotland

**Keywords:** crystal structure, bipyridinium cations, polynitrile anions, mol­ecular conformation, hydrogen bonding

## Abstract

In 2,2′-bipyridin-1-ium 1,1,3,3-tetra­cyano-2-eth­oxy­prop-2-en-1-ide, C_10_H_9_N_2_^+^·C_9_H_5_N_4_O^−^, (I), the ethyl group in the anion is disordered over two sets of atomic sites with occupancies 0.634 (9) and 0.366 (9), and the dihedral angle between the ring planes in the cation is 2.11 (7)°. The two independent C(CN)_2_ groups in the anion make dihedral angles of 10.60 (6) and 12.44 (4)° with the central propenide unit, and the bond distances in the anion provide evidence for extensive electronic delocalization. In bis­(2,2′-bipyridin-1-ium) 1,1,3,3-tetra­cyano-2-(di­cyano­methyl­ene)propane-1,3-diide [alternative name bis­(2,2′-bipyridin-1-ium) tris­(di­cyano­methyl­ene)methane­diide], 2C_10_H_9_N_2_^+^·C_10_N_6_^2−^ (II), the dihedral angles between the ring planes in the two independent cations are 7.7 (2) and 10.92 (17)°. The anion exhibits approximate *C*_3_ symmetry, consistent with extensive electronic delocalization, and the three independent C(CN)_2_ groups make dihedral angles of 23.8 (2), 27.0 (3) and 27.4 (2)° with the central plane. The ions in (I) are linked by an N—H⋯N hydrogen bond and the resulting ion pairs are linked by two independent C—H⋯N hydrogen bonds, forming a ribbon containing alternating *R*_4_^4^(18) and *R*_4_^4^(26) rings, where both ring types are centrosymmetric. The ions in (II) are linked by two independent N—H⋯N hydrogen bonds and the resulting ion triplets are linked by a C—H⋯N hydrogen bond, forming a *C*_2_^1^(7) chain containing anions and only one type of cation, with the other cation linked to the chain by a further C—H⋯N hydrogen bond.

## Abstract 2

In each of the title compounds, the anion shows evidence of extensive electronic delocalization. A combination of N—H⋯N and *X*—H⋯N hydrogen bonds links the ions in (I) into a ribbon of alternating centrosymmetric 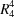
(18) and 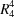
(26) rings, and those in (II) into simple 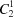
(7) chains of alternating cations and anion with further cations pendent from the chain.

## Chemical context   

Polynitrile anions have received considerable attention recently because of their importance in both coordination chemistry and in mol­ecular materials chemistry (Miyazaki *et al.*, 2003 ▸; Batten & Murray, 2003 ▸; Benmansour *et al.*, 2007 ▸; Setifi, Domasevitch *et al.*, 2013 ▸; Setifi, Setifi *et al.*, 2013 ▸; Setifi, Lehchili *et al.*, 2014 ▸). These organic anions are inter­esting for their extensive electronic delocalization, and for their structural versatility, in particular the potential to utilize a variety of coordination modes, including their action as bridging ligands between metal centres in μ_2_-, μ_3_- or μ_4_- modes, so forming polymeric assemblies which can be one-, two- or three-dimensional. Thus such anions readily form binary complexes with transition-metal and ternary complexes in which a transition-metal centre is also coordinated by other bridging or chelating ligands, and such materials exhibit inter­esting magnetic properties (Atmani *et al.*, 2008 ▸; Benmansour *et al.*, 2008 ▸, 2010 ▸, 2012 ▸; Setifi *et al.*, 2009 ▸).
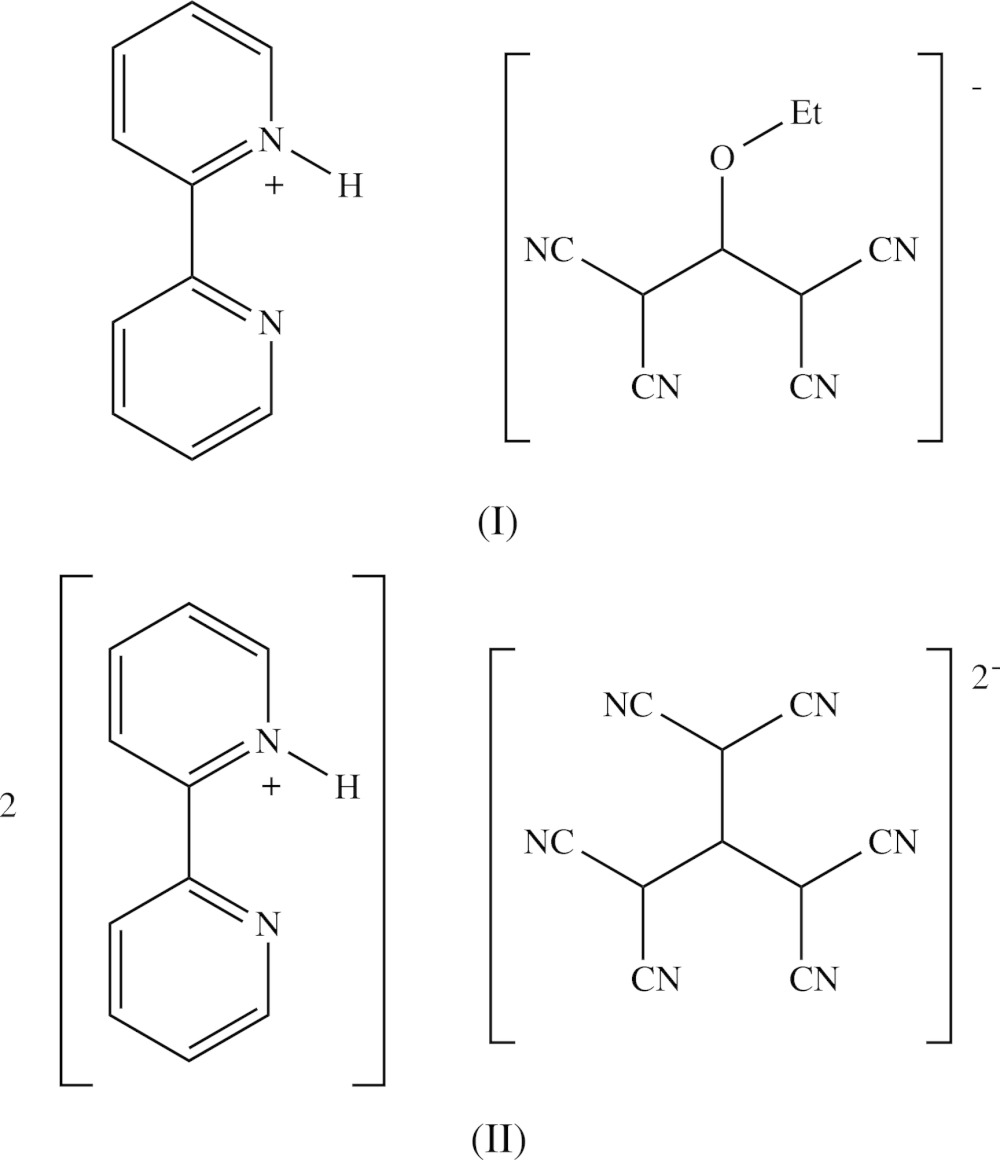


In view of the possible roles of these versatile anionic ligands, we have been inter­ested in using them in combination with other chelating or bridging neutral co-ligands to explore their structural and electronic characteristics in the extensive field of mol­ecular materials exhibiting the spin-crossover (SCO) phenomenon (Dupouy *et al.*, 2008 ▸, 2009 ▸; Setifi, Charles *et al.*, 2014 ▸). During the course of attempts to prepare such complexes, using the anions 1,1,3,3-tetra­cyano-2-eth­oxy­propenide (tcnoet) and tris­(di­cyano­methyl­ene)methane­diide (tcpd), we isolated the two title compounds whose structures are described here.

## Structural commentary   

**Figure 1:**
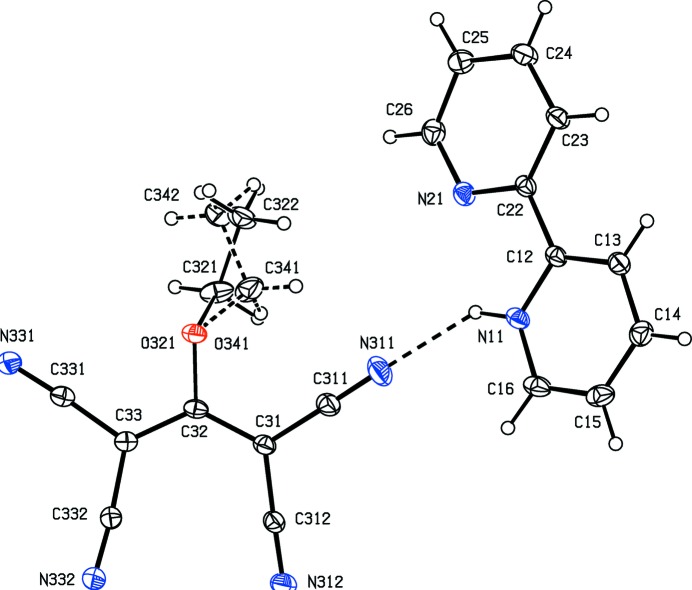
The independent ionic components of compound (I) showing the atom-labelling scheme and the N—H⋯N hydrogen bond within the selected asymmetric unit. Displacement ellipsoids are drawn at the 30% probability level.

**Figure 2:**
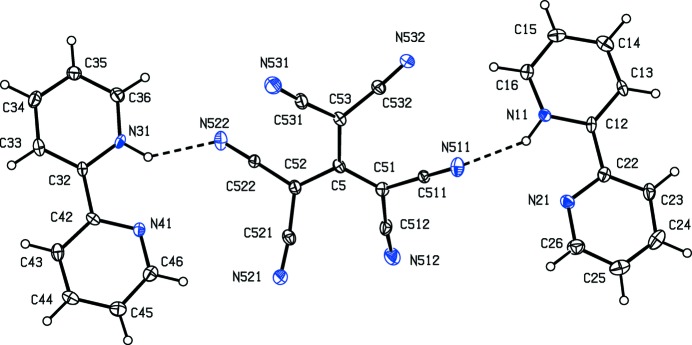
The independent ionic components of compound (II) showing the atom-labelling scheme and the N—H⋯N hydrogen bonds within the selected asymmetric unit. Displacement ellipsoids are drawn at the 30% probability level.

Compound (I) consists of a 2,2′-bipyridin-1-ium cation and a 1,1,3,3-tetra­cyano-2-eth­oxy­propenide anion in which the C atoms of the ethyl group are disordered over two sets of sites having occupancies 0.634 (9) and 0.366 (9). In the selected asymmetric unit for (I) (Fig. 1 ▸) the two ions are linked by an N—H⋯N hydrogen bond (Table 1 ▸). For compound (II), which consists of two 2,2′-bipyridin-1-ium cations and a single tris­(di­cyano­methyl­ene)methane­diide dianion, it was possible to select an asymmetric unit (Fig. 2 ▸) in which the two cations are both linked to the anion by N—H⋯N hydrogen bonds (Table 2 ▸), although an asymmetric unit selected in this way does not fit neatly into the reference unit cell. It will be convenient to refer to the cations of compound (II) containing the atoms N11 and N31 as cations of types 1 and 2 respectively.

In none of the cations are the two rings exactly parallel: the dihedral angle between the mean planes of the two rings in the cation of compound (I) is 2.11 (7)°, and the corresponding angles for the type 1 and 2 cations of compound (II) are 10.92 (17) and 7.7 (2)° respectively. Although each cation contains a short intra-cation N—H⋯N contact (Tables 1 ▸ and 2 ▸), the very small N—H⋯N angles indicate that these contacts are unlikely to be of structural significance (*cf.* Wood *et al.*, 2009 ▸).

In the anion of compound (I), the central bonds C31—C32 and C32—C33 have lengths which are equal within experimental uncertainly (Table 3 ▸). In addition, the four C—C bonds linking the cyano substituents to the central propenide unit are not only similar in length, but all of them are short for their type [mean value (Allen *et al.*, 1987 ▸) 1.431 Å, lower quartile value 1.425 Å]; on the other hand, the C—N distances are all similar and long for their type (mean value 1.136 Å, upper quartile value 1.142 Å). These observations point to extensive delocalization of the negative charge in the anion of (I) with the forms (*A*)–(*F*) (see scheme below) all playing a role in the overall electronic structure. Accordingly, the N—H⋯N hydrogen bond linking the two ions within the selected asymmetric unit of (I) is a charge-assisted hydrogen bond (Gilli *et al.*, 1994 ▸). The tetra­cyano­propenide fragment of this anion is not planar: the two C(CN)_2_ units are twisted out of the plane of the central C_3_O core in a conrotatory fashion, and the dihedral angles between the planes of the C(CN)_2_ units and that of the central core are 10.60 (6)° and 12.44 (4)° respectively for the two units containing atoms C31 and C33.
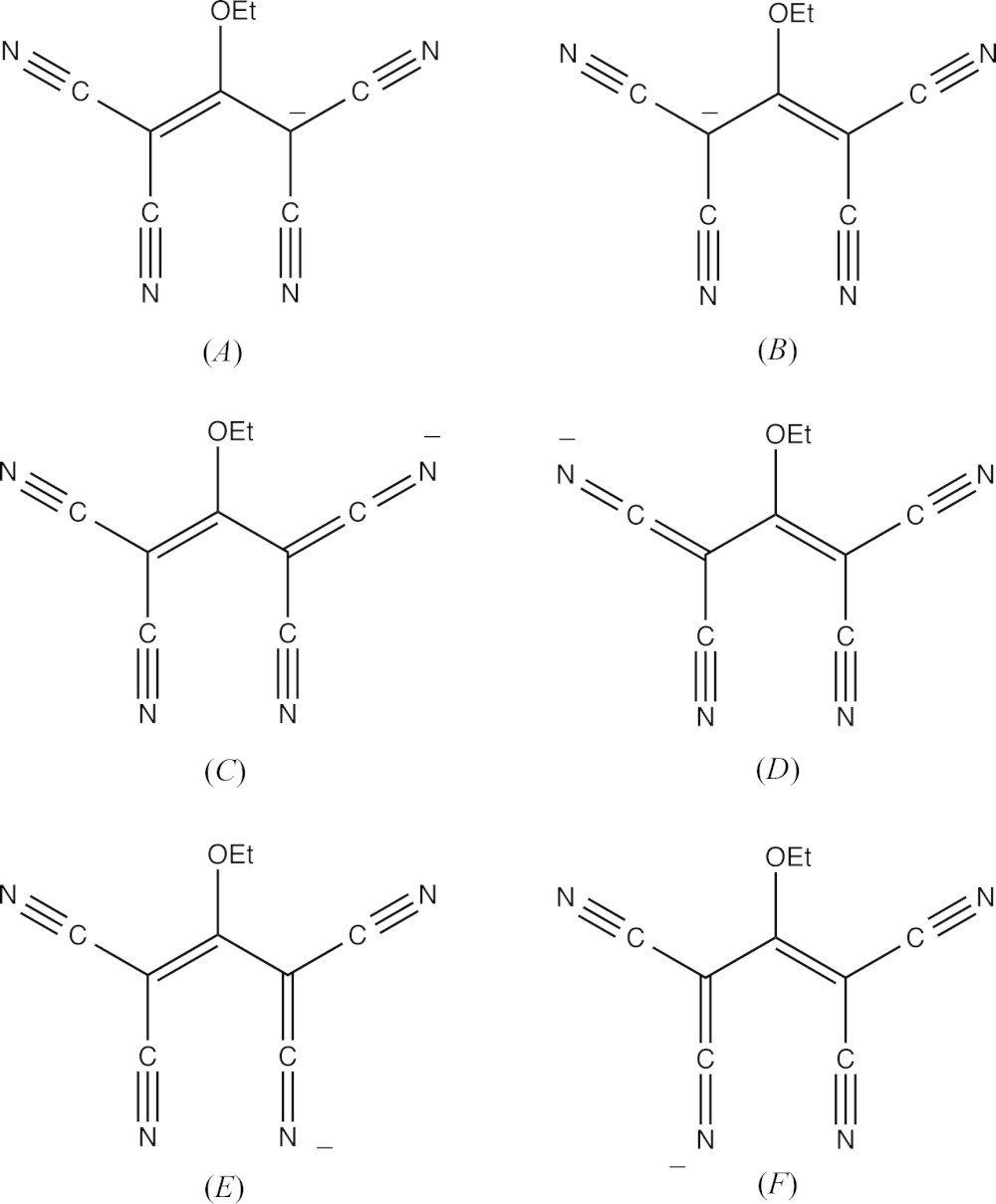


In the anion of compound (II), the geometry at the central atom C5 (Fig. 2 ▸) is planar, and the three C—C bonds involving atom C5 are similar in length (Table 4 ▸). Each of the independent C(CN)_2_ units is rotated out of the plane of the central four-atom core, with dihedral angles between the planes of these three units and that of the central core of 23.8 (3), 27.0 (3) and 27.4 (2)°, respectively, for the C(CN)_2_ units containing atoms C51, C52 and C53. These rotations are in a concerted sense, giving approximate mol­ecular, but not crystallographic, symmetry of *D*_3_ (32) type for the anion. Although the bond distances involving the cyano substituents show some variations (Table 4 ▸) the approximate overall *D*_3_ symmetry is consistent with delocalization of the two negative charges over the whole anion, particularly into the cyano groups.

## Supra­molecular inter­actions   

**Figure 3:**
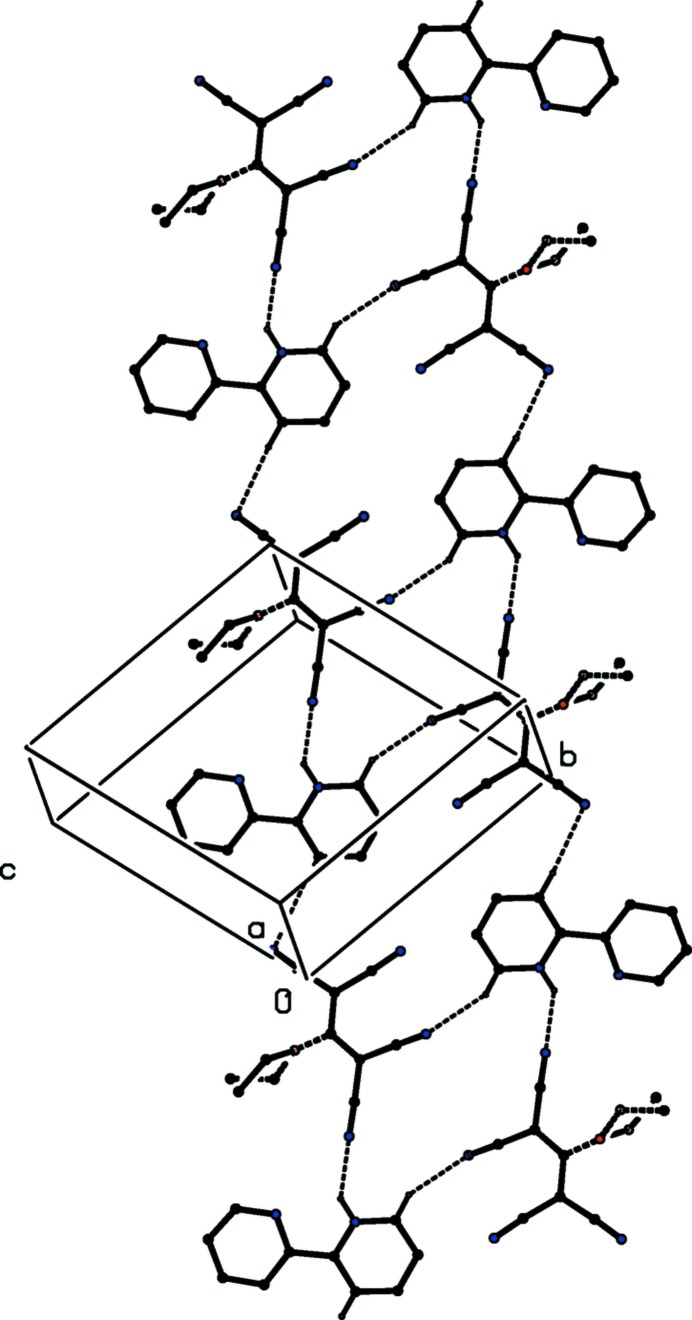
Part of the crystal structure of compound (I) showing the formation of a hydrogen-bonded ribbon parallel to [111] in which centrosymmetric 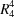
(18) and 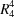
(26) rings alternate. For the sake of clarity, H atoms not involved in the motifs shown have been omitted.

**Figure 4:**
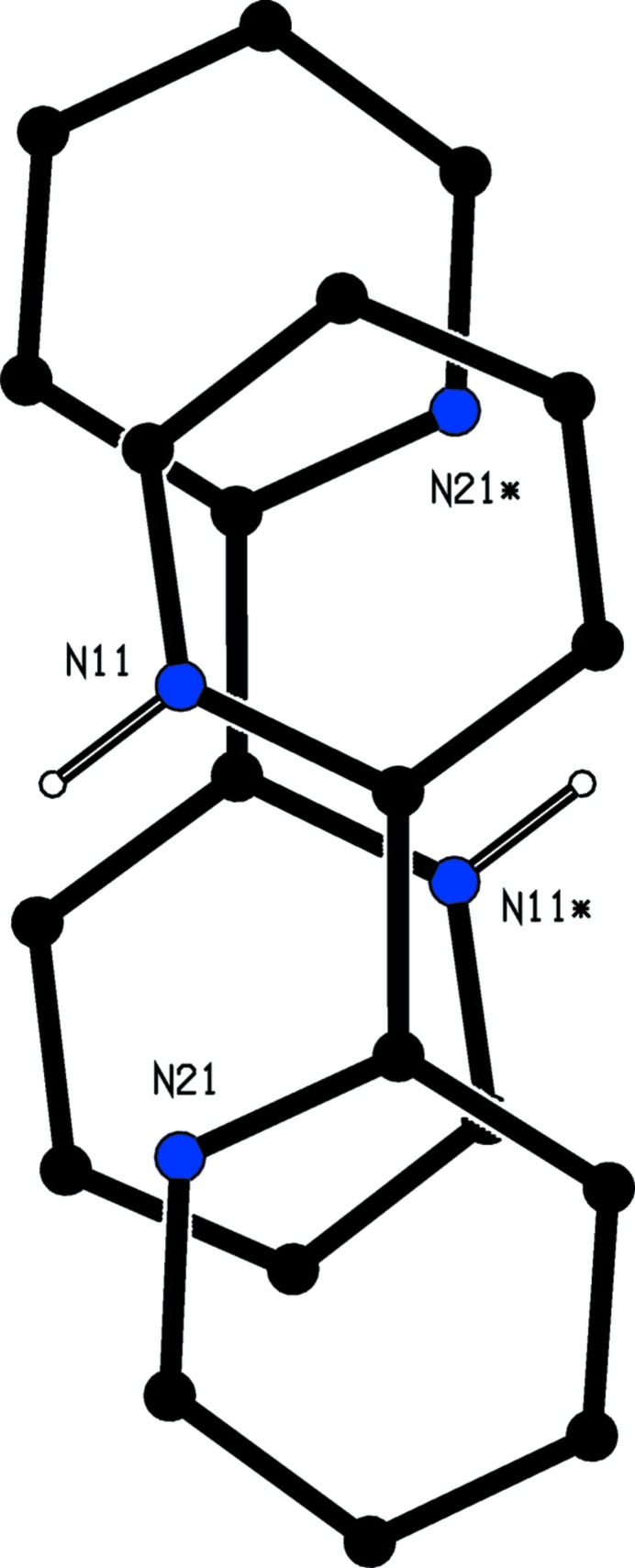
Part of the crystal structure of compound (I) showing the overlap between pairs of inversion-related cations, viewed normal to the ring planes. For the sake of clarity, the unit-cell outline, the anions, and H atoms bonded to C atoms in the cations have all been omitted. Atoms marked with an asterisk (*) are at the symmetry position (−*x*, 1 − *y*, 1 − *z*).

The supra­molecular assembly in compound (I) is determined by the linkage of the ion pairs, themselves inter­nally linked by an N—H⋯N hydrogen bond (Fig. 1 ▸), by two independent C—H⋯N hydrogen bonds both of which involve donors in the protonated pyridyl ring (Table 1 ▸), and both of which therefore can be regarded as charge-assisted hydrogen bonds. The hydrogen bond having atom C13 as the donor links ion pairs related by translation, forming a 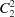
(12) (Bernstein *et al.*, 1995 ▸) chain running parallel to the [111] direction (Fig. 3 ▸). The hydrogen bond having atom C16 as the donor links ion pairs related by inversion, forming a centrosymmetric 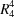
(18) motif (Fig. 3 ▸). The combination of these two inter­actions generates a ribbon running parallel to [111] in which 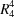
(18) rings centred at (*n* − ½, *n*, *n* − ½) alternate with 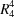
(26) rings centred at (*n*, *n* + ½, *n*), where *n* represents an integer in both cases (Fig. 3 ▸). A single ribbon of this type passes through each unit cell. The crystal structure of compound (I) contains no C—H⋯π hydrogen bonds, but there is a single rather weak π–π stacking inter­action between components of adjacent ribbons. The planes of the protonated pyridyl ring of the reference cation and of the unprotonated ring of the cation at (−*x*, 1 − *y*, 1 − *z*) make a dihedral angle of 2.11 (7)°: the ring-centroid separation is 3.7395 (8) Å and the shortest perpendicular distance from the centroid of one ring to the plane of the other is 3.3413 (5) Å, corresponding to a ring-centroid offset of *ca* 1.65 Å, so that there is only a very modest overlap of the two rings in question (Fig. 4 ▸). If this inter­action is regarded as structurally significant, its effect is to link the ribbons (Fig. 3 ▸) into a sheet parallel to (1
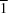
0).

**Figure 5:**
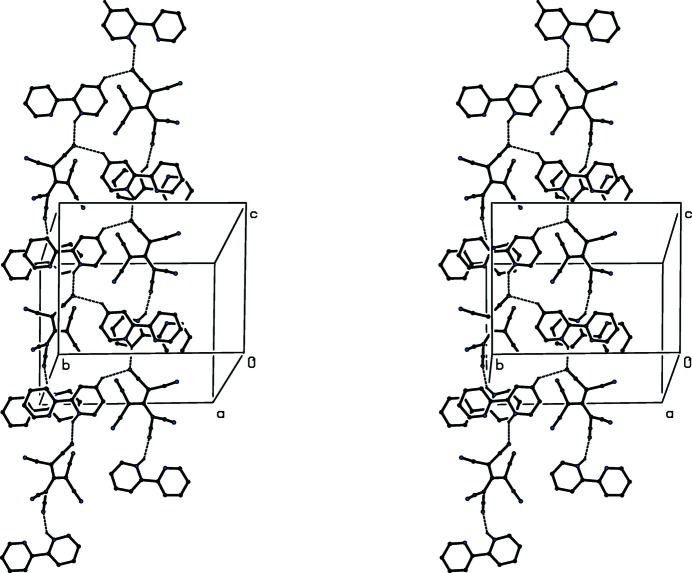
A stereoview of part of the crystal structure of compound (II) showing the formation of a hydrogen-bonded 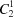
(7) chain parallel to [001] from which the type 1 cations are pendent. For the sake of clarity, the H atoms not involved in the motifs shown have been omitted.

Despite the presence of three independent ions in the structure of compound (II), the supra­molecular assembly in (II) is somewhat simpler than that in (I). Ion triplets (Fig. 2 ▸) which are related by the *c*-glide plane at *y* = 0.75 are linked by a C—H⋯N hydrogen bond (Table 2 ▸), forming a 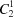
(7) chain running parallel to the [001] direction (Fig. 5 ▸). This chain comprises alternating anions and type 2 cations, while the type 1 cations are simply pendent from the chain. Two chains of this type, related to one another by inversion, pass through each unit cell but there are no direction-specific inter­actions between adjacent chains. Hydrogen bonds of the C—H⋯π type are absent from the crystal structure of compound (II) and the only π–π stacking inter­action lies within the hydrogen-bonded chain.

## Database survey   

We have recently reported the structures of several salts containing the 2-eth­oxy-1,1,3,3-tetra­cyano­propenide anion, including salts with the bis­(2,2′-bi-1*H*-imidazole)­copper(II) cation (Gaamoune *et al.*, 2010 ▸), with tris­(phen­an­thro­line)iron(II) (Setifi, Setifi *et al.*, 2013 ▸), with the 1,1′-diethyl-4,4′-bi­pyridine-1,1′-diium dication (Setifi, Lehchili *et al.*, 2014 ▸) and with tris­(2,2′-bi­pyridine)­iron(II) (Setifi, Setifi *et al.*, 2014 ▸). In each of these salts, the cyano substituents in the anion adopt a very similar conformation to that observed here in compound (I) with, in each case, a similar pattern of bond distances and hence of electronic delocalization. Despite the disparate nature of the counter-ions, the anion conformation is almost constant, suggesting that this is determined primarily by intra-anion forces, rather than by inter-ion inter­actions.

The structures of two organic salts containing the 2-di­cyano­methyl­ene-1,1,3,3-tetra­cyaopropenediide anion have been reported. In both the *N*,*N*′-dimethyl-4,4–bipyridindiium salt [CSD (Groom & Allen, 2014 ▸) refcode BELTER; Nakamura *et al.*, 1981 ▸)] and the bis­(quinolinium) salt (CSD refcode QUCNPR10; Sakanoue *et al.*, 1971 ▸) the anion adopts a conformation having approximately *D*_3_ symmetry, just as found in compound (II) reported here: indeed, the anion in QUCNPR10 lies across a twofold rotation axis in space group *Pbcn*, so that while two of the twofold rotation axes are only approximate, the third is a crystallographic axis. As in compound (II), the C—C and C—N distances in the anions in both BELTER and QUCNPR10 show a degree of variation, but again the approximate symmetry is consistent with extensive electronic delocalization. The structures of the isomorphous salts of this anion with the cations [Ca(H_2_O)_6_]^2+^ (CSD refcode CAHCYB; Bekoe *et al.*, 1967 ▸) and [Ba(H_2_O)_6_]^2+^ (CSD refcode BACMCP; Bekoe *et al.*, 1963 ▸) have been determined, but no atomic coordinates are deposited in the CSD. A number of salts containing the 2,2′-bipyridin-1-ium cation with a range of organic anions have been structurally analysed, but more relevant to the present study are three salts of this cation with simple inorganic anions. In the hydrated monobromide (Bowen *et al.*, 2004 ▸), the bromide ions and the water mol­ecules are linked by O—H⋯Br hydrogen bonds, forming 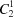
(4) chains to which the cations are linked by N—H⋯ O hydrogen bonds. In the thio­cyanate salt, in which the cations are disordered over two sets of atomic sites (Kavitha *et al.*, 2006 ▸), the ions are linked by a combination of N—H⋯N and C—H⋯N hydrogen bonds, forming 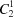
(6) chains, while in the hydrogensulfate salt a combination of five independent hydrogen bonds links the ions into complex sheets (Kavitha *et al.*, 2006 ▸).

## Synthesis and crystallization   

The salts K(tcnoet) and K_2_(tcpd) were prepared using published methods (Middleton *et al.*, 1958 ▸; Middleton & Engelhardt, 1958 ▸). Compounds (I) and (II) were prepared under solvothermal conditions in Teflon-lined steel autoclaves (inner volume *ca* 30 cm^3^). For the synthesis of salt (I), a mixture of iron(II) sulfate hepta­hydrate (28 mg, 0.1 mmol), 2,2′-bi­pyridine (16 mg, 0.1 mmol) and Ktcnoet (45 mg, 0.2 mmol) was dissolved in water–ethanol (4:1 *v*/*v*, 15 cm^3^) and then held in the autoclave at 393 K for 3 d. After slowly cooling to room temperature, pale-orange crystals of (I) suitable for single-crystal X-ray diffraction were obtained (yield 15%). The synthesis of (II) was similar to that of (I), but using K_2_tcpd (50 mg, 0.2 mmol) instead of K(tcnoet), giving yellow crystals suitable for single-crystal X-ray diffraction (yield 40%).

## Refinement   

Crystal data, data collection and structure refinement details are summarized in Table 5 ▸. All H atoms in the cations were located in difference maps. The H atoms bonded to C atoms in the cations were then treated as riding atoms in geometrically idealized positions with C—H distances 0.95 Å and *U*_iso_(H) = 1.2*U*_eq_(C): for H atoms bonded to N atoms, the atomic coordinates were refined with *U*_iso_(H) = 1.2*U*_eq_(N), giving the N—H distances shown in Tables 1 ▸ and 2 ▸. It was apparent from an early stage that the eth­oxy substituent in the anion of compound (I) was disordered over two sets of atomic sites having unequal occupancy. For the minor occupancy component, atoms O341, C341 and C342 (see Fig. 1 ▸), the bonded distances and the one angle non-bonded distances were constrained to be identical to the corresponding distances in the major component, atoms O321, C321 and C322, subject to s.u. values of 0.005 and 0.01 Å respectively. In addition, the atomic coordinates and anisotropic displacement parameters of atoms O321 and O341 were constrained to be identical. Subject to these conditions, the site occupancies refined to values of 0.634 (9) and 0.366 (9). The H atoms in the disordered ethyl group of the anion in compound (I) were included in calculated positions with C—H distances of 0.98 Å with *U*_iso_(H) = 1.5*U*_eq_(C) for the methyl groups, which were permitted to rotate but not to tilt, and C—H distances of 0.99 Å with *U*_iso_(H) = 1.2*U*_eq_(C) for the CH_2_ groups.

## Supplementary Material

Crystal structure: contains datablock(s) global, I, II. DOI: 10.1107/S2056989015007306/hb7404sup1.cif

Structure factors: contains datablock(s) I. DOI: 10.1107/S2056989015007306/hb7404Isup2.hkl

Structure factors: contains datablock(s) II. DOI: 10.1107/S2056989015007306/hb7404IIsup3.hkl

Supporting information file. DOI: 10.1107/S2056989015007306/hb7404Isup4.cml

Supporting information file. DOI: 10.1107/S2056989015007306/hb7404IIsup5.cml

CCDC references: 1059034, 1059033

Additional supporting information:  crystallographic information; 3D view; checkCIF report

## Figures and Tables

**Table 1:** Hydrogen-bond geometry (Å, °) for (I)

*D*—H⋯*A*	*D*—H	H⋯*A*	*D*⋯*A*	*D*—H⋯*A*
N11—H11⋯N21	0.901 (15)	2.202 (15)	2.6306 (15)	108.5 (12)
N11—H11⋯N311	0.901 (15)	2.082 (15)	2.8268 (17)	139.2 (13)
C13—H13⋯N331^i^	0.95	2.52	3.4294 (18)	160
C16—H16⋯N312^ii^	0.95	2.38	3.2238 (18)	148

Symmetry codes: (i) 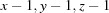; (ii) 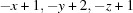.

**Table 2:** Hydrogen-bond geometry (Å, °) for (II)

*D*—H⋯*A*	*D*—H	H⋯*A*	*D*⋯*A*	*D*—H⋯*A*
N11—H11⋯N21	0.91 (3)	2.15 (3)	2.621 (4)	111 (3)
N11—H11⋯N511	0.91 (3)	2.08 (4)	2.874 (5)	145 (3)
N31—H31⋯N41	0.91 (4)	2.14 (3)	2.627 (4)	113 (3)
N31—H31⋯N522	0.91 (4)	2.15 (4)	2.888 (5)	138 (3)
C16—H16⋯N532	0.95	2.56	3.472 (6)	162
C34—H34⋯N522^i^	0.95	2.62	3.391 (5)	139

Symmetry code: (i) 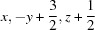.

**Table 3:** Selected geometric parameters (Å, °) for (I)

C31—C32	1.3982 (17)	C32—O321	1.3618 (13)
C32—C33	1.3956 (16)	O321—C321	1.428 (2)
C31—C311	1.4136 (16)	C311—N311	1.1471 (17)
C31—C312	1.4224 (16)	C312—N312	1.1498 (16)
C33—C331	1.4261 (17)	C331—N331	1.1504 (16)
C33—C332	1.4181 (16)	C332—N332	1.1522 (16)
			
C32—C31—C311	119.84 (11)	C32—C33—C331	119.94 (10)
C32—C31—C312	123.31 (10)	C32—C33—C332	124.72 (11)
C311—C31—C312	116.80 (11)	C331—C33—C332	115.15 (10)
N311—C311—C31	178.44 (17)	C31—C32—C33	127.46 (10)
N312—C312—C31	178.53 (13)	O321—C32—C31	118.45 (10)
N331—C331—C33	176.77 (13)	O321—C32—C33	114.02 (10)
N332—C332—C33	175.54 (13)		
			
C31—C32—C33—C331	−171.92 (11)	C31—C32—O321—C321	76.5 (3)
C31—C32—C33—C332	13.3 (2)	C33—C32—O321—C321	−106.2 (3)
C33—C32—C31—C311	−166.68 (12)	C32—O321—C321—C322	−156.1 (4)
C33—C32—C31—C312	10.92 (19)		

**Table 4:** Selected geometric parameters (Å, °) for (II)

C5—C51	1.411 (5)	C53—C532	1.437 (6)
C5—C52	1.413 (5)	C511—N511	1.136 (4)
C5—C53	1.433 (5)	C512—N512	1.140 (5)
C51—C511	1.413 (5)	C521—N521	1.155 (5)
C51—C512	1.439 (5)	C522—N522	1.153 (5)
C52—C521	1.428 (5)	C531—N531	1.129 (5)
C52—C522	1.410 (5)	C532—N532	1.121 (5)
C53—C531	1.428 (6)		
			
C51—C5—C52	122.1 (3)	C5—C52—C521	121.9 (3)
C51—C5—C53	119.5 (3)	C5—C52—C522	123.0 (3)
C52—C5—C53	118.4 (4)	C521—C52—C522	115.0 (3)
C5—C51—C511	120.9 (3)	C5—C53—C531	121.2 (4)
C5—C51—C512	122.0 (3)	C5—C53—C532	122.0 (4)
C511—C51—C512	117.1 (3)	C531—C53—C532	116.9 (3)
			
C51—C5—C52—C521	26.5 (6)	C51—C5—C53—C531	−153.1 (4)
C51—C5—C52—C522	−150.5 (4)	C51—C5—C53—C532	25.9 (6)
C52—C5—C53—C531	28.8 (6)	C52—C5—C51—C511	−156.5 (4)
C52—C5—C53—C532	−152.2 (4)	C52—C5—C51—C512	22.0 (6)
C53—C5—C51—C511	25.5 (6)	C53—C5—C52—C521	−155.5 (4)
C53—C5—C51—C512	−156.0 (4)	C53—C5—C52—C522	27.5 (6)

**Table 5:** Experimental details

	(I)	(II)
Crystal data		
Chemical formula	C_10_H_9_N_2_^+^·C_9_H_5_N_4_O^−^	2C_10_H_9_N_2_^+^·C_10_N_6_^2−^
*M*_r_	342.36	518.54
Crystal system, space group	Triclinic, *P* 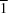	Monoclinic, *P*2_1_/*c*
Temperature (K)	123	173
*a*, *b*, *c* (Å)	7.2514 (1), 10.6647 (2), 11.5619 (2)	13.4195 (8), 16.1801 (8), 12.9058 (9)
α, β, γ (°)	100.020 (1), 104.372 (1), 92.590 (1)	90, 116.721 (3), 90
*V* (Å^3^)	849.27 (3)	2503.0 (3)
*Z*	2	4
Radiation type	Mo *K*α	Mo *K*α
μ (mm^−1^)	0.09	0.09
Crystal size (mm)	0.40 × 0.35 × 0.13	0.21 × 0.14 × 0.09

Data collection		
Diffractometer	Bruker APEXII CCD	Bruker APEXII CCD
Absorption correction	Multi-scan (*SADABS*; Sheldrick, 2003 ▸)	–
*T*_min_, *T*_max_	0.870, 0.988	–
No. of measured, independent and observed [*I* > 2σ(*I*)] reflections	6234, 4152, 3447	14513, 4607, 2137
*R*_int_	0.017	0.086
(sin θ/λ)_max_ (Å^−1^)	0.667	0.603

Refinement		
*R*[*F* ^2^ > 2σ(*F* ^2^)], *wR*(*F* ^2^), *S*	0.042, 0.103, 1.02	0.067, 0.183, 0.98
No. of reflections	4152	4607
No. of parameters	259	367
No. of restraints	3	0
H-atom treatment	H atoms treated by a mixture of independent and constrained refinement	H atoms treated by a mixture of independent and constrained refinement
Δρ_max_, Δρ_min_ (e Å^−3^)	0.25, −0.20	0.38, −0.26

Computer programs: *COLLECT* (Bruker, 2008 ▸), *DENZO-SMN* (Otwinowski & Minor, 1997 ▸), *APEX2* and *SAINT* (Bruker, 2009 ▸, *SIR2011* (Burla *et al.*, 2012 ▸), *SHELXS97* (Sheldrick, 2008 ▸), *SHELXL2014* (Sheldrick, 2015 ▸) and *PLATON* (Spek, 2009 ▸).

## supplementary crystallographic information

### (I) 2,2'-Bipyridin-1-ium 1,1,3,3-tetracyano-2-ethoxyprop-2-en-1-ide Crystal data

**Table:**

C_10_H_9_N_2_^+^·C_9_H_5_N_4_O^−^	*Z* = 2
*M**_r_* = 342.36	*F*(000) = 356
Triclinic, *P*1	*D*_x_ = 1.339 Mg m^−^^3^
*a* = 7.2514 (1) Å	Mo *K*α radiation, λ = 0.71073 Å
*b* = 10.6647 (2) Å	Cell parameters from 4152 reflections
*c* = 11.5619 (2) Å	θ = 2.9–28.3°
α = 100.020 (1)°	µ = 0.09 mm^−^^1^
β = 104.372 (1)°	*T* = 123 K
γ = 92.590 (1)°	Plate, pale orange
*V* = 849.27 (3) Å^3^	0.40 × 0.35 × 0.13 mm

### (I) 2,2'-Bipyridin-1-ium 1,1,3,3-tetracyano-2-ethoxyprop-2-en-1-ide Data collection

**Table:**

Bruker APEXII CCD diffractometer	4152 independent reflections
Radiation source: fine-focus sealed tube	3447 reflections with *I* > 2σ(*I*)
Graphite monochromator	*R*_int_ = 0.017
φ & ω scans	θ_max_ = 28.3°, θ_min_ = 2.9°
Absorption correction: multi-scan (SADABS; Sheldrick, 2003)	*h* = −9→9
*T*_min_ = 0.870, *T*_max_ = 0.988	*k* = −11→14
6234 measured reflections	*l* = −15→15

### (I) 2,2'-Bipyridin-1-ium 1,1,3,3-tetracyano-2-ethoxyprop-2-en-1-ide Refinement

**Table:**

Refinement on *F*^2^	3 restraints
Least-squares matrix: full	Hydrogen site location: mixed
*R*[*F*^2^ > 2σ(*F*^2^)] = 0.042	H atoms treated by a mixture of independent and constrained refinement
*wR*(*F*^2^) = 0.103	*w* = 1/[σ^2^(*F*_o_^2^) + (0.0362*P*)^2^ + 0.2911*P*] where *P* = (*F*_o_^2^ + 2*F*_c_^2^)/3
*S* = 1.02	(Δ/σ)_max_ < 0.001
4152 reflections	Δρ_max_ = 0.25 e Å^−^^3^
259 parameters	Δρ_min_ = −0.20 e Å^−^^3^

### (I) 2,2'-Bipyridin-1-ium 1,1,3,3-tetracyano-2-ethoxyprop-2-en-1-ide Special details

**Table:**

Geometry. All e.s.d.'s (except the e.s.d. in the dihedral angle between two l.s. planes) are estimated using the full covariance matrix. The cell e.s.d.'s are taken into account individually in the estimation of e.s.d.'s in distances, angles and torsion angles; correlations between e.s.d.'s in cell parameters are only used when they are defined by crystal symmetry. An approximate (isotropic) treatment of cell e.s.d.'s is used for estimating e.s.d.'s involving l.s. planes.

### (I) 2,2'-Bipyridin-1-ium 1,1,3,3-tetracyano-2-ethoxyprop-2-en-1-ide Fractional atomic coordinates and isotropic or equivalent isotropic displacement parameters (Å^2^)

**Table:**

	*x*	*y*	*z*	*U*_iso_*/*U*_eq_	Occ. (<1)
N11	0.21857 (15)	0.52707 (9)	0.43771 (10)	0.0285 (2)	
H11	0.304 (2)	0.5526 (14)	0.5105 (14)	0.034*	
C12	0.13048 (16)	0.40769 (10)	0.41532 (11)	0.0250 (2)	
C13	−0.00916 (18)	0.36846 (12)	0.30713 (11)	0.0300 (3)	
H13	−0.0760	0.2858	0.2891	0.036*	
C14	−0.0511 (2)	0.45071 (13)	0.22510 (12)	0.0354 (3)	
H14	−0.1463	0.4240	0.1505	0.042*	
C15	0.0455 (2)	0.57160 (13)	0.25183 (13)	0.0370 (3)	
H15	0.0191	0.6278	0.1956	0.044*	
C16	0.18018 (19)	0.60851 (12)	0.36102 (13)	0.0347 (3)	
H16	0.2459	0.6917	0.3820	0.042*	
N21	0.33192 (15)	0.39123 (10)	0.60741 (9)	0.0298 (2)	
C22	0.19143 (16)	0.33195 (11)	0.51119 (10)	0.0248 (2)	
C23	0.10772 (18)	0.20973 (12)	0.50218 (12)	0.0316 (3)	
H23	0.0098	0.1704	0.4324	0.038*	
C24	0.1711 (2)	0.14670 (12)	0.59796 (13)	0.0358 (3)	
H24	0.1167	0.0631	0.5949	0.043*	
C25	0.31365 (19)	0.20658 (13)	0.69753 (12)	0.0336 (3)	
H25	0.3585	0.1656	0.7645	0.040*	
C26	0.39027 (18)	0.32811 (13)	0.69780 (12)	0.0334 (3)	
H26	0.4898	0.3686	0.7662	0.040*	
C31	0.63575 (17)	0.92545 (11)	0.75796 (11)	0.0258 (2)	
C32	0.66992 (16)	0.94146 (11)	0.88432 (11)	0.0243 (2)	
C33	0.73776 (16)	1.05351 (11)	0.97022 (10)	0.0252 (2)	
C311	0.52991 (19)	0.81335 (12)	0.68277 (12)	0.0327 (3)	
N311	0.4472 (2)	0.72078 (12)	0.62292 (12)	0.0525 (4)	
C312	0.69653 (17)	1.01940 (11)	0.69840 (11)	0.0278 (3)	
N312	0.74430 (19)	1.09353 (11)	0.64791 (11)	0.0396 (3)	
C331	0.78777 (17)	1.04922 (11)	1.09671 (11)	0.0281 (3)	
N331	0.83067 (17)	1.05186 (11)	1.19987 (10)	0.0365 (3)	
C332	0.74824 (17)	1.17768 (11)	0.94263 (11)	0.0277 (2)	
N332	0.75833 (18)	1.28170 (10)	0.92815 (10)	0.0366 (3)	
O321	0.62639 (12)	0.83982 (8)	0.93268 (8)	0.0279 (2)	0.634 (9)
C321	0.7550 (4)	0.7422 (3)	0.9381 (5)	0.0381 (9)	0.634 (9)
H32A	0.8632	0.7663	1.0117	0.046*	0.634 (9)
H32B	0.8069	0.7308	0.8658	0.046*	0.634 (9)
C322	0.6481 (8)	0.6213 (3)	0.9417 (6)	0.0430 (11)	0.634 (9)
H32C	0.7343	0.5530	0.9458	0.064*	0.634 (9)
H32D	0.5422	0.5977	0.8681	0.064*	0.634 (9)
H32E	0.5974	0.6334	1.0135	0.064*	0.634 (9)
O341	0.62639 (12)	0.83982 (8)	0.93268 (8)	0.0279 (2)	0.366 (9)
C341	0.6787 (17)	0.7134 (5)	0.8864 (6)	0.051 (2)	0.366 (9)
H34A	0.8019	0.7218	0.8647	0.061*	0.366 (9)
H34B	0.5796	0.6712	0.8125	0.061*	0.366 (9)
C342	0.6957 (17)	0.6360 (6)	0.9825 (6)	0.0423 (18)	0.366 (9)
H42C	0.7350	0.5519	0.9541	0.063*	0.366 (9)
H42D	0.5718	0.6252	1.0010	0.063*	0.366 (9)
H42E	0.7914	0.6796	1.0560	0.063*	0.366 (9)

### (I) 2,2'-Bipyridin-1-ium 1,1,3,3-tetracyano-2-ethoxyprop-2-en-1-ide Atomic displacement parameters (Å^2^)

**Table:**

	*U*^11^	*U*^22^	*U*^33^	*U*^12^	*U*^13^	*U*^23^
N11	0.0302 (5)	0.0213 (5)	0.0365 (6)	−0.0010 (4)	0.0157 (4)	0.0026 (4)
C12	0.0269 (6)	0.0197 (5)	0.0310 (6)	0.0016 (4)	0.0148 (5)	0.0016 (4)
C13	0.0346 (6)	0.0245 (6)	0.0316 (6)	0.0005 (5)	0.0123 (5)	0.0021 (5)
C14	0.0414 (7)	0.0367 (7)	0.0310 (6)	0.0075 (6)	0.0142 (6)	0.0065 (5)
C15	0.0456 (8)	0.0347 (7)	0.0422 (7)	0.0120 (6)	0.0257 (6)	0.0161 (6)
C16	0.0394 (7)	0.0243 (6)	0.0491 (8)	0.0029 (5)	0.0261 (6)	0.0097 (5)
N21	0.0296 (5)	0.0285 (5)	0.0301 (5)	−0.0028 (4)	0.0097 (4)	0.0011 (4)
C22	0.0254 (5)	0.0216 (5)	0.0283 (6)	0.0006 (4)	0.0113 (4)	0.0012 (4)
C23	0.0338 (6)	0.0232 (6)	0.0342 (6)	−0.0026 (5)	0.0047 (5)	0.0035 (5)
C24	0.0398 (7)	0.0245 (6)	0.0419 (7)	−0.0003 (5)	0.0076 (6)	0.0084 (5)
C25	0.0326 (6)	0.0342 (7)	0.0361 (7)	0.0068 (5)	0.0092 (5)	0.0107 (5)
C26	0.0293 (6)	0.0373 (7)	0.0313 (6)	−0.0007 (5)	0.0066 (5)	0.0031 (5)
C31	0.0275 (6)	0.0206 (5)	0.0310 (6)	−0.0001 (4)	0.0110 (5)	0.0049 (4)
C32	0.0212 (5)	0.0218 (5)	0.0332 (6)	0.0027 (4)	0.0102 (4)	0.0092 (4)
C33	0.0241 (5)	0.0246 (6)	0.0280 (6)	0.0020 (4)	0.0067 (4)	0.0082 (4)
C311	0.0382 (7)	0.0266 (6)	0.0371 (7)	−0.0017 (5)	0.0197 (5)	0.0027 (5)
N311	0.0676 (9)	0.0374 (7)	0.0510 (8)	−0.0208 (6)	0.0312 (7)	−0.0117 (6)
C312	0.0327 (6)	0.0219 (5)	0.0297 (6)	0.0019 (4)	0.0116 (5)	0.0020 (4)
N312	0.0552 (7)	0.0269 (5)	0.0423 (6)	0.0002 (5)	0.0233 (6)	0.0075 (5)
C331	0.0272 (6)	0.0240 (6)	0.0334 (7)	−0.0001 (4)	0.0071 (5)	0.0082 (5)
N331	0.0447 (7)	0.0315 (6)	0.0314 (6)	−0.0005 (5)	0.0047 (5)	0.0095 (4)
C332	0.0308 (6)	0.0257 (6)	0.0253 (6)	0.0025 (4)	0.0057 (5)	0.0042 (4)
N332	0.0522 (7)	0.0265 (6)	0.0300 (6)	0.0033 (5)	0.0082 (5)	0.0063 (4)
O321	0.0309 (4)	0.0215 (4)	0.0357 (5)	0.0025 (3)	0.0145 (4)	0.0092 (3)
C321	0.0313 (13)	0.0412 (15)	0.055 (2)	0.0185 (11)	0.0194 (12)	0.0291 (14)
C322	0.061 (3)	0.0208 (13)	0.054 (3)	0.0070 (12)	0.027 (2)	0.0080 (15)
O341	0.0309 (4)	0.0215 (4)	0.0357 (5)	0.0025 (3)	0.0145 (4)	0.0092 (3)
C341	0.090 (6)	0.037 (3)	0.039 (3)	0.033 (3)	0.029 (3)	0.019 (2)
C342	0.061 (5)	0.026 (3)	0.043 (4)	0.010 (2)	0.018 (3)	0.007 (2)

### (I) 2,2'-Bipyridin-1-ium 1,1,3,3-tetracyano-2-ethoxyprop-2-en-1-ide Geometric parameters (Å, º)

**Table:**

N11—C16	1.3361 (17)	C32—C33	1.3956 (16)
N11—C12	1.3512 (14)	C31—C311	1.4136 (16)
N11—H11	0.901 (16)	C31—C312	1.4224 (16)
C12—C13	1.3842 (17)	C33—C331	1.4261 (17)
C12—C22	1.4755 (17)	C33—C332	1.4181 (16)
C13—C14	1.3900 (18)	C32—O321	1.3618 (13)
C13—H13	0.9500	O321—C321	1.428 (2)
C14—C15	1.3860 (19)	C311—N311	1.1471 (17)
C14—H14	0.9500	C312—N312	1.1498 (16)
C15—C16	1.372 (2)	C331—N331	1.1504 (16)
C15—H15	0.9500	C332—N332	1.1522 (16)
C16—H16	0.9500	C321—C322	1.487 (3)
N21—C26	1.3325 (17)	C321—H32A	0.9900
N21—C22	1.3451 (15)	C321—H32B	0.9900
C22—C23	1.3886 (16)	C322—H32C	0.9800
C23—C24	1.3870 (18)	C322—H32D	0.9800
C23—H23	0.9500	C322—H32E	0.9800
C24—C25	1.3767 (19)	C341—C342	1.480 (4)
C24—H24	0.9500	C341—H34A	0.9900
C25—C26	1.3861 (18)	C341—H34B	0.9900
C25—H25	0.9500	C342—H42C	0.9800
C26—H26	0.9500	C342—H42D	0.9800
C31—C32	1.3982 (17)	C342—H42E	0.9800
			
C16—N11—C12	123.83 (12)	C32—C31—C312	123.31 (10)
C16—N11—H11	119.5 (10)	C311—C31—C312	116.80 (11)
C12—N11—H11	116.6 (10)	N311—C311—C31	178.44 (17)
N11—C12—C13	117.84 (11)	N312—C312—C31	178.53 (13)
N11—C12—C22	116.02 (11)	N331—C331—C33	176.77 (13)
C13—C12—C22	126.13 (10)	N332—C332—C33	175.54 (13)
C12—C13—C14	119.62 (12)	C32—C33—C331	119.94 (10)
C12—C13—H13	120.2	C32—C33—C332	124.72 (11)
C14—C13—H13	120.2	C331—C33—C332	115.15 (10)
C15—C14—C13	120.18 (13)	C31—C32—C33	127.46 (10)
C15—C14—H14	119.9	O321—C32—C31	118.45 (10)
C13—C14—H14	119.9	O321—C32—C33	114.02 (10)
C16—C15—C14	118.72 (12)	C32—O321—C321	117.18 (14)
C16—C15—H15	120.6	O321—C321—C322	108.2 (3)
C14—C15—H15	120.6	O321—C321—H32A	110.1
N11—C16—C15	119.78 (12)	C322—C321—H32A	110.1
N11—C16—H16	120.1	O321—C321—H32B	110.1
C15—C16—H16	120.1	C322—C321—H32B	110.1
C26—N21—C22	117.27 (11)	H32A—C321—H32B	108.4
N21—C22—C23	123.22 (11)	C321—C322—H32C	109.5
N21—C22—C12	114.70 (10)	C321—C322—H32D	109.5
C23—C22—C12	122.08 (11)	H32C—C322—H32D	109.5
C24—C23—C22	118.13 (12)	C321—C322—H32E	109.5
C24—C23—H23	120.9	H32C—C322—H32E	109.5
C22—C23—H23	120.9	H32D—C322—H32E	109.5
C25—C24—C23	119.32 (12)	C342—C341—H34A	110.0
C25—C24—H24	120.3	C342—C341—H34B	110.0
C23—C24—H24	120.3	H34A—C341—H34B	108.4
C24—C25—C26	118.46 (12)	C341—C342—H42C	109.5
C24—C25—H25	120.8	C341—C342—H42D	109.5
C26—C25—H25	120.8	H42C—C342—H42D	109.5
N21—C26—C25	123.59 (12)	C341—C342—H42E	109.5
N21—C26—H26	118.2	H42C—C342—H42E	109.5
C25—C26—H26	118.2	H42D—C342—H42E	109.5
C32—C31—C311	119.84 (11)		
			
C16—N11—C12—C13	−0.79 (17)	C22—C23—C24—C25	−0.2 (2)
C16—N11—C12—C22	−179.72 (10)	C23—C24—C25—C26	−0.6 (2)
N11—C12—C13—C14	1.28 (17)	C22—N21—C26—C25	−0.31 (18)
C22—C12—C13—C14	−179.92 (11)	C24—C25—C26—N21	0.9 (2)
C12—C13—C14—C15	−0.39 (19)	C311—C31—C32—O321	10.27 (17)
C13—C14—C15—C16	−1.02 (19)	C312—C31—C32—O321	−172.13 (11)
C12—N11—C16—C15	−0.64 (18)	O321—C32—C33—C331	11.01 (16)
C14—C15—C16—N11	1.53 (19)	O321—C32—C33—C332	−163.73 (11)
C26—N21—C22—C23	−0.53 (17)	C31—C32—C33—C331	−171.92 (11)
C26—N21—C22—C12	178.83 (10)	C31—C32—C33—C332	13.3 (2)
N11—C12—C22—N21	−1.88 (15)	C33—C32—C31—C311	−166.68 (12)
C13—C12—C22—N21	179.30 (11)	C33—C32—C31—C312	10.92 (19)
N11—C12—C22—C23	177.49 (11)	C31—C32—O321—C321	76.5 (3)
C13—C12—C22—C23	−1.34 (18)	C33—C32—O321—C321	−106.2 (3)
N21—C22—C23—C24	0.76 (19)	C32—O321—C321—C322	−156.1 (4)
C12—C22—C23—C24	−178.54 (11)		

### (I) 2,2'-Bipyridin-1-ium 1,1,3,3-tetracyano-2-ethoxyprop-2-en-1-ide Hydrogen-bond geometry (Å, º)

**Table:**

*D*—H···*A*	*D*—H	H···*A*	*D*···*A*	*D*—H···*A*
N11—H11···N21	0.901 (15)	2.202 (15)	2.6306 (15)	108.5 (12)
N11—H11···N311	0.901 (15)	2.082 (15)	2.8268 (17)	139.2 (13)
C13—H13···N331^i^	0.95	2.52	3.4294 (18)	160
C16—H16···N312^ii^	0.95	2.38	3.2238 (18)	148

Symmetry codes: (i) *x*−1, *y*−1, *z*−1; (ii) −*x*+1, −*y*+2, −*z*+1.

### (II) Bis(2,2'-bipyridin-1-ium) 1,1,3,3-tetracyano-2-(dicyanomethylene)propane-1,3-diide Crystal data

**Table:**

2C_10_H_9_N_2_^+^·C_10_N_6_^2^^−^	*F*(000) = 1072
*M**_r_* = 518.54	*D*_x_ = 1.376 Mg m^−^^3^
Monoclinic, *P*2_1_/*c*	Mo *K*α radiation, λ = 0.71073 Å
*a* = 13.4195 (8) Å	Cell parameters from 5568 reflections
*b* = 16.1801 (8) Å	θ = 1.7–28.3°
*c* = 12.9058 (9) Å	µ = 0.09 mm^−^^1^
β = 116.721 (3)°	*T* = 173 K
*V* = 2503.0 (3) Å^3^	Block, yellow
*Z* = 4	0.21 × 0.14 × 0.09 mm

### (II) Bis(2,2'-bipyridin-1-ium) 1,1,3,3-tetracyano-2-(dicyanomethylene)propane-1,3-diide Data collection

**Table:**

Bruker APEXII CCD diffractometer	2137 reflections with *I* > 2σ(*I*)
Radiation source: fine-focus sealed tube	*R*_int_ = 0.086
Graphite monochromator	θ_max_ = 25.4°, θ_min_ = 1.7°
φ & ω scans	*h* = −13→16
14513 measured reflections	*k* = −18→19
4607 independent reflections	*l* = −15→14

### (II) Bis(2,2'-bipyridin-1-ium) 1,1,3,3-tetracyano-2-(dicyanomethylene)propane-1,3-diide Refinement

**Table:**

Refinement on *F*^2^	0 restraints
Least-squares matrix: full	Hydrogen site location: mixed
*R*[*F*^2^ > 2σ(*F*^2^)] = 0.067	H atoms treated by a mixture of independent and constrained refinement
*wR*(*F*^2^) = 0.183	*w* = 1/[σ^2^(*F*_o_^2^) + (0.0713*P*)^2^] where *P* = (*F*_o_^2^ + 2*F*_c_^2^)/3
*S* = 0.98	(Δ/σ)_max_ < 0.001
4607 reflections	Δρ_max_ = 0.38 e Å^−^^3^
367 parameters	Δρ_min_ = −0.26 e Å^−^^3^

### (II) Bis(2,2'-bipyridin-1-ium) 1,1,3,3-tetracyano-2-(dicyanomethylene)propane-1,3-diide Special details

**Table:**

Geometry. All e.s.d.'s (except the e.s.d. in the dihedral angle between two l.s. planes) are estimated using the full covariance matrix. The cell e.s.d.'s are taken into account individually in the estimation of e.s.d.'s in distances, angles and torsion angles; correlations between e.s.d.'s in cell parameters are only used when they are defined by crystal symmetry. An approximate (isotropic) treatment of cell e.s.d.'s is used for estimating e.s.d.'s involving l.s. planes.

### (II) Bis(2,2'-bipyridin-1-ium) 1,1,3,3-tetracyano-2-(dicyanomethylene)propane-1,3-diide Fractional atomic coordinates and isotropic or equivalent isotropic displacement parameters (Å^2^)

**Table:**

	*x*	*y*	*z*	*U*_iso_*/*U*_eq_	
N11	0.3903 (2)	0.52309 (19)	0.3636 (3)	0.0206 (8)	
H11	0.383 (3)	0.483 (2)	0.409 (3)	0.025*	
C12	0.3951 (3)	0.4944 (2)	0.2684 (3)	0.0209 (9)	
C13	0.4011 (3)	0.5512 (2)	0.1917 (3)	0.0245 (10)	
H13	0.4047	0.5330	0.1234	0.029*	
C14	0.4019 (3)	0.6344 (3)	0.2146 (4)	0.0320 (11)	
H14	0.4044	0.6737	0.1612	0.038*	
C15	0.3990 (3)	0.6611 (2)	0.3151 (4)	0.0317 (11)	
H15	0.4006	0.7184	0.3317	0.038*	
C16	0.3937 (3)	0.6040 (2)	0.3895 (4)	0.0250 (10)	
H16	0.3925	0.6210	0.4593	0.030*	
N21	0.3977 (3)	0.36187 (19)	0.3483 (3)	0.0273 (9)	
C22	0.3892 (3)	0.4033 (2)	0.2542 (4)	0.0235 (10)	
C23	0.3719 (3)	0.3648 (2)	0.1524 (4)	0.0300 (11)	
H23	0.3679	0.3958	0.0882	0.036*	
C24	0.3604 (3)	0.2798 (3)	0.1462 (4)	0.0401 (12)	
H24	0.3465	0.2513	0.0766	0.048*	
C25	0.3694 (4)	0.2373 (3)	0.2413 (5)	0.0409 (12)	
H25	0.3622	0.1789	0.2392	0.049*	
C26	0.3890 (3)	0.2808 (3)	0.3404 (4)	0.0369 (12)	
H26	0.3966	0.2507	0.4067	0.044*	
N31	0.1131 (3)	0.62657 (19)	1.1404 (3)	0.0246 (9)	
H31	0.115 (3)	0.592 (2)	1.086 (3)	0.030*	
C32	0.1142 (3)	0.5861 (2)	1.2326 (3)	0.0185 (9)	
C33	0.1241 (3)	0.6335 (2)	1.3253 (4)	0.0285 (10)	
H33	0.1266	0.6075	1.3924	0.034*	
C34	0.1307 (3)	0.7188 (2)	1.3214 (4)	0.0329 (11)	
H34	0.1378	0.7510	1.3859	0.039*	
C35	0.1269 (3)	0.7569 (2)	1.2248 (4)	0.0292 (11)	
H35	0.1295	0.8154	1.2209	0.035*	
C36	0.1195 (3)	0.7089 (2)	1.1343 (4)	0.0316 (11)	
H36	0.1188	0.7339	1.0674	0.038*	
N41	0.1140 (3)	0.46447 (19)	1.1296 (3)	0.0227 (8)	
C42	0.1077 (3)	0.4958 (2)	1.2227 (3)	0.0207 (9)	
C43	0.0985 (3)	0.4463 (2)	1.3062 (4)	0.0283 (10)	
H43	0.0916	0.4702	1.3699	0.034*	
C44	0.0998 (3)	0.3618 (2)	1.2944 (4)	0.0307 (11)	
H44	0.0965	0.3264	1.3515	0.037*	
C45	0.1060 (3)	0.3294 (3)	1.1987 (4)	0.0299 (11)	
H45	0.1053	0.2713	1.1875	0.036*	
C46	0.1132 (3)	0.3834 (2)	1.1198 (4)	0.0285 (11)	
H46	0.1178	0.3608	1.0542	0.034*	
C5	0.2470 (3)	0.5068 (2)	0.7478 (3)	0.0208 (9)	
C51	0.2195 (3)	0.4600 (2)	0.6463 (3)	0.0236 (10)	
C511	0.2894 (3)	0.4602 (2)	0.5908 (3)	0.0200 (9)	
N511	0.3457 (3)	0.4592 (2)	0.5464 (3)	0.0344 (9)	
C512	0.1214 (4)	0.4089 (3)	0.5963 (4)	0.0290 (11)	
N512	0.0434 (3)	0.3695 (2)	0.5487 (3)	0.0435 (11)	
C52	0.2057 (3)	0.4868 (2)	0.8279 (3)	0.0255 (10)	
C521	0.1746 (3)	0.4044 (3)	0.8405 (4)	0.0271 (10)	
N521	0.1522 (3)	0.3395 (2)	0.8607 (3)	0.0375 (10)	
C522	0.1872 (3)	0.5464 (2)	0.8970 (4)	0.0233 (10)	
N522	0.1686 (3)	0.5942 (2)	0.9524 (3)	0.0351 (9)	
C53	0.3161 (3)	0.5786 (2)	0.7695 (4)	0.0271 (10)	
C531	0.3839 (4)	0.6070 (3)	0.8849 (4)	0.0304 (11)	
N531	0.4417 (3)	0.6318 (2)	0.9736 (3)	0.0456 (11)	
C532	0.3220 (3)	0.6245 (2)	0.6771 (4)	0.0261 (10)	
N532	0.3242 (3)	0.6642 (2)	0.6070 (3)	0.0331 (9)	

### (II) Bis(2,2'-bipyridin-1-ium) 1,1,3,3-tetracyano-2-(dicyanomethylene)propane-1,3-diide Atomic displacement parameters (Å^2^)

**Table:**

	*U*^11^	*U*^22^	*U*^33^	*U*^12^	*U*^13^	*U*^23^
N11	0.0184 (19)	0.023 (2)	0.023 (2)	−0.0027 (15)	0.0117 (17)	−0.0006 (16)
C12	0.015 (2)	0.035 (2)	0.014 (2)	−0.0003 (17)	0.0069 (19)	−0.0038 (19)
C13	0.024 (3)	0.036 (3)	0.017 (2)	−0.0011 (19)	0.012 (2)	0.004 (2)
C14	0.023 (3)	0.037 (3)	0.031 (3)	−0.001 (2)	0.007 (2)	0.010 (2)
C15	0.028 (3)	0.022 (2)	0.042 (3)	0.0000 (18)	0.013 (2)	0.001 (2)
C16	0.020 (2)	0.030 (2)	0.023 (3)	0.0050 (18)	0.009 (2)	0.000 (2)
N21	0.031 (2)	0.025 (2)	0.027 (2)	0.0023 (15)	0.0142 (19)	0.0049 (16)
C22	0.015 (2)	0.030 (2)	0.025 (3)	0.0011 (18)	0.010 (2)	−0.003 (2)
C23	0.027 (3)	0.043 (3)	0.025 (3)	−0.005 (2)	0.016 (2)	−0.006 (2)
C24	0.032 (3)	0.045 (3)	0.040 (3)	0.005 (2)	0.012 (3)	−0.016 (2)
C25	0.034 (3)	0.030 (3)	0.055 (4)	−0.003 (2)	0.017 (3)	−0.006 (3)
C26	0.041 (3)	0.029 (3)	0.037 (3)	0.001 (2)	0.014 (3)	0.001 (2)
N31	0.035 (2)	0.022 (2)	0.022 (2)	0.0014 (15)	0.0167 (19)	−0.0069 (16)
C32	0.015 (2)	0.028 (2)	0.014 (2)	0.0015 (17)	0.007 (2)	−0.0003 (19)
C33	0.030 (3)	0.039 (3)	0.021 (3)	0.006 (2)	0.015 (2)	0.004 (2)
C34	0.035 (3)	0.038 (3)	0.033 (3)	−0.005 (2)	0.021 (2)	−0.013 (2)
C35	0.033 (3)	0.025 (2)	0.039 (3)	0.0009 (18)	0.024 (2)	0.000 (2)
C36	0.040 (3)	0.028 (3)	0.035 (3)	0.002 (2)	0.024 (3)	0.003 (2)
N41	0.023 (2)	0.026 (2)	0.020 (2)	−0.0006 (15)	0.0112 (17)	−0.0023 (16)
C42	0.018 (2)	0.022 (2)	0.023 (3)	−0.0001 (16)	0.009 (2)	0.0065 (19)
C43	0.023 (3)	0.042 (3)	0.018 (3)	0.0018 (19)	0.007 (2)	0.000 (2)
C44	0.030 (3)	0.029 (3)	0.033 (3)	−0.0034 (19)	0.013 (2)	0.008 (2)
C45	0.022 (3)	0.031 (2)	0.035 (3)	−0.0032 (18)	0.011 (2)	0.000 (2)
C46	0.021 (3)	0.033 (3)	0.031 (3)	−0.0029 (18)	0.011 (2)	−0.007 (2)
C5	0.016 (2)	0.027 (2)	0.023 (2)	−0.0003 (16)	0.0113 (19)	0.0029 (18)
C51	0.020 (2)	0.026 (2)	0.024 (3)	0.0013 (18)	0.010 (2)	0.000 (2)
C511	0.021 (2)	0.022 (2)	0.020 (2)	−0.0016 (17)	0.011 (2)	−0.0003 (18)
N511	0.029 (2)	0.049 (2)	0.028 (2)	0.0044 (17)	0.015 (2)	−0.0028 (18)
C512	0.030 (3)	0.036 (3)	0.028 (3)	−0.002 (2)	0.019 (2)	−0.007 (2)
N512	0.047 (3)	0.051 (2)	0.038 (3)	−0.017 (2)	0.023 (2)	−0.005 (2)
C52	0.032 (3)	0.025 (2)	0.023 (3)	0.0061 (18)	0.016 (2)	0.0042 (19)
C521	0.023 (3)	0.035 (3)	0.024 (3)	−0.004 (2)	0.012 (2)	0.001 (2)
N521	0.050 (3)	0.035 (2)	0.032 (3)	−0.0127 (18)	0.022 (2)	−0.0073 (18)
C522	0.028 (3)	0.024 (2)	0.019 (2)	−0.0046 (18)	0.012 (2)	−0.003 (2)
N522	0.038 (2)	0.046 (2)	0.025 (2)	0.0089 (19)	0.017 (2)	0.0015 (19)
C53	0.032 (3)	0.028 (2)	0.025 (3)	−0.0022 (19)	0.017 (2)	0.003 (2)
C531	0.034 (3)	0.037 (3)	0.023 (3)	−0.005 (2)	0.015 (3)	0.000 (2)
N531	0.050 (3)	0.052 (2)	0.031 (3)	−0.023 (2)	0.014 (2)	−0.008 (2)
C532	0.024 (3)	0.029 (2)	0.026 (3)	−0.0022 (18)	0.012 (2)	0.002 (2)
N532	0.043 (2)	0.028 (2)	0.035 (3)	−0.0075 (17)	0.024 (2)	−0.0010 (18)

### (II) Bis(2,2'-bipyridin-1-ium) 1,1,3,3-tetracyano-2-(dicyanomethylene)propane-1,3-diide Geometric parameters (Å, º)

**Table:**

N11—C12	1.342 (4)	C34—H34	0.9500
N11—C16	1.346 (5)	C35—C36	1.368 (6)
N11—H11	0.91 (4)	C35—H35	0.9500
C12—C13	1.379 (5)	C36—H36	0.9500
C12—C22	1.483 (5)	N41—C46	1.317 (5)
C13—C14	1.378 (5)	N41—C42	1.341 (5)
C13—H13	0.9500	C42—C43	1.392 (5)
C14—C15	1.384 (6)	C43—C44	1.375 (5)
C14—H14	0.9500	C43—H43	0.9500
C15—C16	1.357 (5)	C44—C45	1.379 (6)
C15—H15	0.9500	C44—H44	0.9500
C16—H16	0.9500	C45—C46	1.378 (5)
N21—C26	1.316 (5)	C45—H45	0.9500
N21—C22	1.346 (5)	C46—H46	0.9500
C22—C23	1.375 (5)	C5—C51	1.411 (5)
C23—C24	1.383 (6)	C5—C52	1.413 (5)
C23—H23	0.9500	C5—C53	1.433 (5)
C24—C25	1.364 (6)	C51—C511	1.413 (5)
C24—H24	0.9500	C51—C512	1.439 (5)
C25—C26	1.378 (6)	C52—C521	1.428 (5)
C25—H25	0.9500	C52—C522	1.410 (5)
C26—H26	0.9500	C53—C531	1.428 (6)
N31—C36	1.340 (5)	C53—C532	1.437 (6)
N31—C32	1.353 (5)	C511—N511	1.136 (4)
N31—H31	0.90 (4)	C512—N512	1.140 (5)
C32—C33	1.375 (5)	C521—N521	1.155 (5)
C32—C42	1.466 (5)	C522—N522	1.153 (5)
C33—C34	1.386 (5)	C531—N531	1.129 (5)
C33—H33	0.9500	C532—N532	1.121 (5)
C34—C35	1.371 (6)		
			
C12—N11—C16	123.5 (3)	C35—C34—C33	120.3 (4)
C12—N11—H11	114 (2)	C35—C34—H34	119.9
C16—N11—H11	123 (2)	C33—C34—H34	119.9
N11—C12—C13	118.0 (4)	C36—C35—C34	118.7 (4)
N11—C12—C22	115.8 (3)	C36—C35—H35	120.7
C13—C12—C22	126.2 (4)	C34—C35—H35	120.7
C14—C13—C12	119.6 (4)	N31—C36—C35	119.8 (4)
C14—C13—H13	120.2	N31—C36—H36	120.1
C12—C13—H13	120.2	C35—C36—H36	120.1
C13—C14—C15	120.4 (4)	C46—N41—C42	117.5 (3)
C13—C14—H14	119.8	N41—C42—C43	122.6 (4)
C15—C14—H14	119.8	N41—C42—C32	115.4 (3)
C16—C15—C14	118.9 (4)	C43—C42—C32	121.9 (4)
C16—C15—H15	120.6	C44—C43—C42	118.5 (4)
C14—C15—H15	120.6	C44—C43—H43	120.8
N11—C16—C15	119.6 (4)	C42—C43—H43	120.8
N11—C16—H16	120.2	C43—C44—C45	119.1 (4)
C15—C16—H16	120.2	C43—C44—H44	120.5
C26—N21—C22	117.3 (4)	C45—C44—H44	120.5
N21—C22—C23	123.0 (4)	C46—C45—C44	118.2 (4)
N21—C22—C12	113.9 (3)	C46—C45—H45	120.9
C23—C22—C12	123.0 (4)	C44—C45—H45	120.9
C22—C23—C24	118.2 (4)	N41—C46—C45	124.1 (4)
C22—C23—H23	120.9	N41—C46—H46	117.9
C24—C23—H23	120.9	C45—C46—H46	117.9
C25—C24—C23	119.1 (4)	C51—C5—C52	122.1 (3)
C25—C24—H24	120.5	C51—C5—C53	119.5 (3)
C23—C24—H24	120.5	C52—C5—C53	118.4 (4)
C24—C25—C26	118.7 (4)	C5—C51—C511	120.9 (3)
C24—C25—H25	120.6	C5—C51—C512	122.0 (3)
C26—C25—H25	120.6	C511—C51—C512	117.1 (3)
N21—C26—C25	123.6 (4)	N511—C511—C51	179.0 (4)
N21—C26—H26	118.2	N512—C512—C51	174.8 (5)
C25—C26—H26	118.2	C5—C52—C521	121.9 (3)
C36—N31—C32	123.8 (4)	C5—C52—C522	123.0 (3)
C36—N31—H31	123 (2)	C521—C52—C522	115.0 (3)
C32—N31—H31	112 (2)	N521—C521—C52	174.0 (4)
N31—C32—C33	117.0 (4)	N522—C522—C52	177.7 (4)
N31—C32—C42	115.4 (3)	C5—C53—C531	121.2 (4)
C33—C32—C42	127.5 (4)	C5—C53—C532	122.0 (4)
C32—C33—C34	120.4 (4)	C531—C53—C532	116.9 (3)
C32—C33—H33	119.8	N531—C531—C53	175.7 (5)
C34—C33—H33	119.8	N532—C532—C53	176.0 (4)
			
C16—N11—C12—C13	−1.6 (5)	C32—N31—C36—C35	−0.4 (6)
C16—N11—C12—C22	−179.5 (3)	C34—C35—C36—N31	1.7 (6)
N11—C12—C13—C14	−0.1 (5)	C46—N41—C42—C43	−1.2 (5)
C22—C12—C13—C14	177.6 (4)	C46—N41—C42—C32	177.3 (3)
C12—C13—C14—C15	1.4 (6)	N31—C32—C42—N41	7.0 (5)
C13—C14—C15—C16	−1.0 (6)	C33—C32—C42—N41	−171.0 (4)
C12—N11—C16—C15	2.0 (5)	N31—C32—C42—C43	−174.5 (3)
C14—C15—C16—N11	−0.7 (6)	C33—C32—C42—C43	7.4 (6)
C26—N21—C22—C23	0.1 (6)	N41—C42—C43—C44	2.3 (6)
C26—N21—C22—C12	177.5 (3)	C32—C42—C43—C44	−176.1 (4)
N11—C12—C22—N21	−10.0 (5)	C42—C43—C44—C45	−2.3 (6)
C13—C12—C22—N21	172.3 (4)	C43—C44—C45—C46	1.4 (6)
N11—C12—C22—C23	167.4 (3)	C42—N41—C46—C45	0.2 (6)
C13—C12—C22—C23	−10.3 (6)	C44—C45—C46—N41	−0.4 (6)
N21—C22—C23—C24	1.5 (6)	C51—C5—C52—C521	26.5 (6)
C12—C22—C23—C24	−175.8 (3)	C51—C5—C52—C522	−150.5 (4)
C22—C23—C24—C25	−1.6 (6)	C52—C5—C53—C531	28.8 (6)
C23—C24—C25—C26	0.4 (6)	C52—C5—C53—C532	−152.2 (4)
C22—N21—C26—C25	−1.4 (6)	C53—C5—C51—C511	25.5 (6)
C24—C25—C26—N21	1.3 (7)	C53—C5—C51—C512	−156.0 (4)
C36—N31—C32—C33	−1.0 (5)	C51—C5—C53—C531	−153.1 (4)
C36—N31—C32—C42	−179.3 (3)	C51—C5—C53—C532	25.9 (6)
N31—C32—C33—C34	1.1 (5)	C52—C5—C51—C511	−156.5 (4)
C42—C32—C33—C34	179.1 (4)	C52—C5—C51—C512	22.0 (6)
C32—C33—C34—C35	0.2 (6)	C53—C5—C52—C521	−155.5 (4)
C33—C34—C35—C36	−1.6 (6)	C53—C5—C52—C522	27.5 (6)

### (II) Bis(2,2'-bipyridin-1-ium) 1,1,3,3-tetracyano-2-(dicyanomethylene)propane-1,3-diide Hydrogen-bond geometry (Å, º)

**Table:**

*D*—H···*A*	*D*—H	H···*A*	*D*···*A*	*D*—H···*A*
N11—H11···N21	0.91 (3)	2.15 (3)	2.621 (4)	111 (3)
N11—H11···N511	0.91 (3)	2.08 (4)	2.874 (5)	145 (3)
N31—H31···N41	0.91 (4)	2.14 (3)	2.627 (4)	113 (3)
N31—H31···N522	0.91 (4)	2.15 (4)	2.888 (5)	138 (3)
C16—H16···N532	0.95	2.56	3.472 (6)	162
C34—H34···N522^i^	0.95	2.62	3.391 (5)	139

Symmetry code: (i) *x*, −*y*+3/2, *z*+1/2.
